# Everybody's Page

**Published:** 1890-08-23

**Authors:** 


### Everybody's Page.

A chiropodist advertises that he has removed corns from most of the crowned heads of Europe.

Dr. Walker Downie has traced a number of cases of diseases of the pharynx to excess in tea-drinking. Needless to state, most of the cases occurred in women.

Rapid bleaching of linen or cotton fabrics, after washing with soap, is effected, according to Gabriel Dubell, by placing them one hour in a bath containing about one per cent. commercial potassium chlorate, with enough hydrochloric acid to give it a sour taste. The acid is removed by treating with a borax solution, and the fabrics finally well washed with water.

The bodies of several Honveds have lately been found in Hungary, who fought in 1848, and apparently got beaten and took refuge in a disused salt mine. The salt had so thoroughly penetrated the bodies that they were as well preserved as if they had been kept in spirits. In consequence of this discovery experiments will be made in the medical schools of Vienna, Budapest, and Berlin for testing the value of a concentrated solution of salt in preservation of corpses.

The modern child is an awful creature; only a very ignorant aged grandmother will dare to remonstrate or argue in these days, for the child is bound to have the best of it, somewhat in this way:—"You mustn't play with the scissors, dear. I knew a little boy just like you who was playing with a pair of scissors just like that pair, and he put them in his eye, and he put his eye out, and he never could see anything after." The child listened patiently, and said when she got through: "What was the matter with his other eye?"

"Electricity is Life" is the trade mark of a firm that advertises galvanic belts, &c.; Kemmler has found that electricity is death. The subject of execution by electricity has long been before the American public; a long article in its favour appeared in the *Scientific American* in 1873. In 1887 a commission was appointed to investigate and report on the subject. The commission consisted of Messrs. Elbridge T. Gerry, Alfred P. Southwick, and Matthew Hale, who deserve much credit as having fathered and engineered this Bill at Albany and for having brought it to a successful issue. The Bill was passed on June 4th, 1888, and became law on the 1st of January, 1889; the first execution took place on August 5th, 1890. Such is the history of the matter so far. Public opinion is very adverse to the notion of execution by electricity, and it is not probable that it will become common for many years.

Carlyle could use his knowledge of physiology at times to point a moral; in fact, no weapon ever came amiss to his hands. In attacking the *laissez-faire* doctrines in "Past and Present," he says: "Well, if the heart be got round now into the right side, and the liver to the left; if man has no heroism in him deeper than the wish to eat, etc." And in the same volume appears the following delightful story: "A benevolent old surgeon sat once in our company with a patient fallen sick by gourmandizing, whom he had just, too briefly in the patient's judgment, been examining. The foolish patient still at intervals continued to break in on our discourse, which rather promised to take a philosophic turn: 'But I have lost my appetite,' said he, objurgatively, with a tone of irritated pathos; 'I have no appetite; I can't eat!' 'My dear fellow,' answered the doctor in mildest tone, 'it isn't of the slightest consequence;' and continued his philosophic discoursings with us!"

In a curious letter from Arnulf, Bishop of Lisieux, to Pope Alexander III., asking him to dissolve the monastery of Grestain because of certain abuses, it is stated that certain of the monks had killed a sick woman by plunging her into ice-cold water under pretext of working a miraculous cure. Arnulf hints that this was done not so much from ignorance, as from mischief. Not very tender physicians, after all, these holy men of the middle ages.

Many a holiday is spoilt by a sprained ankle, and either subsequent neglect or faulty treatment of the injury. When a sprain occurs let the injured joint be wrapped in flannel dipped in a hot solution of arnica, and cover with surgeons' sheeting; when the pain is quite gone, the ankle or wrist should be held for a few minutes, several times a day, under a tap of cold water. When the swelling has quite gone, if there is a little stiffness on first beginning to use the joint again, hot baths will be found useful. Of course the injured part requires rest.

American doctors are full of consternation at the terrible increase of hysteria amongst their lady patients. With all due deference, we beg to give it as our emphatic opinion that they are solely to blame for the prevalence of this malady, and we ask them if the increase has not been most marked since 1888, when Dr. Louis Fiske-Bryson wrote thus in the *New York Medical Journal*: "A change in environment and mental impression, the introduction of some special pursuit or interest in life, are recommended. *Well-fitting and costly garments are advised?*" Of course every American woman of sense has had hysteria.

Mr. G. A. Rowell, of Oxford, in an essay on "The Beneficent Distribution of the Sense of Pain in the Lower Animals," published some years ago, gives a forcible example of insensibility to pain in a horse. A horse, feeding by the side of the road on Headington Hill, Oxford, had its leg broken by a coach-wheel passing over it just above the fetlock-joint; the bone was dreadfully crushed, and protruded in parts through the skin. Within a few minutes the horse had hobbled to the side of the road, and begun grazing, showing no other signs of pain than holding up the injured leg. It must be a relief to many sensitive men and women to know that the power of feeling pain differs amongst men, and also amongst animals.

Hospital abuse and its remedies are not solely questions of to-day, and those are sanguine persons who think that the present Select Committee of the House of Lords will manage to remedy many of the present evils of our charity system. It was in 1869 that Dr. Mayo was dismissed from St. Bartholomew's Hospital because he refused to see more than fifty new cases a day. The senior students held a crowded meeting and resolved "That this meeting believes that the defects in the present administration of St. Bartholomew's Hospital call for Parliamentary inquiry and permanent Government supervision, in the interests of the public." It was in 1873 that Mr Nelson Hardy, F.R.C.S, wrote his celebrated pamphlet on "Hospital Out-Patient Reform;" it was in 1874 that Dr. John Chapman found the doors of the Metropolitan Free Hospital shut in his face for advocating remedies of existing abuses which are now universally recognized as absolutely necessary. There are some half-dozen medical men who have already been martyrs in the cause; let us hope that at last some good will result, built up on their labours, though the honours will doubtless fall to other men.

